# Brain-Targeted Drug Delivery

**DOI:** 10.3390/pharmaceutics14091835

**Published:** 2022-08-31

**Authors:** Flávia Sousa

**Affiliations:** Adolphe Merkle Institute, University of Fribourg, Chemin des Verdiers 4, 1700 Fribourg, Switzerland; flavia.sousa@unifr.ch

At present, brain diseases affect one in six people worldwide, and they include a wide range of neurological diseases from Alzheimer’s and Parkinson’s diseases to epilepsy, brain injuries, brain cancer, neuroinfections and strokes. The treatment of these diseases is complex and limited due to the presence of the blood–brain barrier (BBB), which covers the entirety of the brain. The BBB not only has the function of protecting the brain from harmful substances but is also a metabolic barrier and a transport regulator of nutrients/serum factors/neurotoxins. Knowing these characteristics when it comes to the treatment of brain diseases makes it easy to understand the lack of efficacy of therapeutic drugs, resulting from the innate resistance of the BBB to permeation. To overcome this limitation, drug delivery systems based on nanotechnology/microtechnology have been wisely developed. Brain-targeted drug delivery allows targeted therapy with a higher therapeutic efficacy and low side effects because it targets moieties present in the drug delivery systems.

Brain-targeted drug delivery research is an active, rich and multidisciplinary research area, and this Special Issue aims to present the current state of the art in the field. A series of nine research articles and three review articles are presented, with authors from 10 different countries, which demonstrates the multidisciplinarity of investigations that have been carried out in this area. This Special Issue brings together the latest research from the treatment of glioblastoma (GBM) to neurodegenerative diseases and epilepsy. Furthermore, literature reviews are presented on the topics of (i) novel drug delivery systems for GBM treatment, (ii) the potential of Alzheimer’s disease immunotherapy, and, lastly, (iii) current methods to detect and monitor macromolecules in the brain.

The main obstacle to treating disorders of the central nervous system (CNS) is the presence of the BBB, which hinders the delivery of therapeutics. It is well known that few small-molecule drugs can cross the BBB, and most biologic drugs cannot. As an alternative route to overcome the BBB, Kouzehgarani et al. evaluated the biodistribution of an anti-EGFR antibody in the rat brain after intra-cisterna magma injection. They show vastly greater and deeper penetration of the monoclonal antibody (mAb) into the brain parenchyma after CSF administration compared to IV administration. The authors demonstrate that circumventing the BBB via CSF microcirculation might be a strategy to improve the delivery of mAbs into the brain, achieving deep penetration of IgG-size biologics [1].

Another administration route that allows us to successfully reach the brain is the intranasal route. Intranasal administration has recently been explored by researchers because it reaches the brain, bypassing the BBB through the olfactory bulb. Petkova et al. used this strategy to enhance gene delivery to the cerebral cortex using hyaluronidase-coated glycol chitosan–DNA polyplexes (GCPH) [2]. The authors show high levels of protein expression in the brain regions upon intranasal administration of hyaluronidase-coated polyplexes. Following the same strategy of intranasal administration, Qizilbash et al. developed a naringenin-encapsulated nanostructured lipid carrier (NGN-NLC) with thymoquinone (TQ) oil to investigate the antidepressant potential of the nanosystem [3]. Their ex vivo and in vivo results show higher penetration and greater antidepressant potential from NGN-NLC compared to NGN suspension achieved by intranasal administration. Lastly, Nathshed et al. developed a pomegranate seed oil (PSO) phospholipid oil gel for nasal administration to test its biological effect on memory and locomotor activity [4]. The results show a significant improvement in the behavior of animals when they were treated with intranasal gel compared to orally administrated oil.

Another common administration route for brain-targeted drug delivery is intracranial. To improve intracranial viral vector delivery in non-human primates (NHPs), Griggs et al. developed a new method based in bench-side convection-enhanced delivery (CED) that provides users with a hands-on CED experience. This method aims to help and guide researchers in the surgical preparations for intracranial viral delivery using CED in NHPs [5]. Indeed, other authors have used CED for the intratumoral administration of biologics. Rechberger et al. studied the effect of direct intratumoral administration of GB-13 in an orthotopic xenograph model of high-grade glioma via CED [6]. This novel peptide–toxin conjugate that binds to IL-13Rα2 was able to significantly decrease tumor size and prolong survival in both diffuse midline and high-grade glioma with high levels of IL-13Rα2, opening doors for IL-13Rα2-targeted therapy.

Focusing on intravenous administration for brain-targeted drug delivery, Nakano et al. developed hollow-gold nanoparticles tethered to liposomes (HGN-liposomes) loaded with muscimol to be released by laser or ultrasound stimulation and to inhibit neurons and suppress epileptiform seizures [7]. The combination of ultrasound stimulation and intravenous administration of HGN-liposomes suppressed seizure activity in the hippocampus, demonstrating the therapeutic potential of HGN-liposomes for controlling epileptiform seizures without continuous exposure.

Drug delivery into the brain can also be done by intraperitoneal administration. Casanova et al. developed PLGA microparticles and nanoparticles loaded with Tolcapone to improve the treatment of Parkinson’s disease (PD) [8]. There is an urgent need to find new and promising therapeutic strategies to treat PD that are able to overcome the BBB. The authors demonstrate that Tolcapone-loaded PLGA nanoparticles were able to revert PD-like symptoms of neurodegeneration in an in vivo model upon intraperitoneal administration.

Undoubtedly, the BBB is essential for protecting the organ from toxins, drugs, and pathogens, serving as a highly selective semipermeable membrane of endothelial cells. Damaging the BBB can lead to serious consequences for brain homeostasis and neuronal degeneration. Yang et al. studied the impact of high-dose acetaminophen (APAP) on the integrity of the BBB, demonstrating increased paracellular permeability of the BBB as well as increased protein expression of claudin-5 in brain microvessels [9]. The authors also observed that APAP-induced paracellular “leak” contributed to increased CNS uptake of codeine, bringing awareness to the biological effects of concomitant administration of APAP with opioids.

Lastly, this Special Issue presents three review articles to provide the reader with a broad overview of brain-targeted drug delivery research. Kheir et al. reviews the literature on the development of new drug delivery systems and novel strategies for GBM treatment [10]. The authors discuss (i) current GBM traditional treatments, (ii) the role of chemokine CXCL12 and its receptor CXCR4 in GBM invasion, (iii) interstitial fluid flow in GBM, (iv) models to study GBM cell migration and (v) innovative treatments for GBM. On the other side, Toshihiko Tashima discusses the potential of Alzheimer’s disease immunotherapy using intravenously administered anti-tau and anti-receptor bispecific antibodies [11]. These anti-tau and bispecific mAbs are able to induce receptor-mediated transcytosis in capillary endothelial cells of the BBB and might represent a solution for Aβ-targeting therapies. Lastly, Custers et al. reviews the literature on the current approaches to detect and monitor macromolecules in the brain directly from the cerebral interstitial fluid [12]. Direct sampling from the cerebral interstitial space can be done via a few techniques such as microdialysis, cerebral open flow microperfusion and electrochemical biosensors. The authors discuss the current limitations and advantages of each technique.

Overall, the articles in this Special Issue highlight a very active and interesting field for society since, in recent years, brain diseases are affecting more people and starting earlier. Therefore, there is an urgent need to understanding the biological properties of the BBB and to apply that knowledge to finding new therapeutic strategies for brain diseases. I would like to thank all the authors of this Special Issue for contributing high-quality works, as well as all the reviewers who critically evaluated the articles. In addition, I would like to thank the Assistant Editor, Mr. Jaimin Tao, for his kind help.
